# SARS-CoV-2 incidence, seroprevalence, and COVID-19 vaccination coverage in the homeless population: a systematic review and meta-analysis

**DOI:** 10.3389/fpubh.2023.1044788

**Published:** 2023-10-10

**Authors:** Yuanhao Liang, Qian Sun, Quanxun Liu, Yulian Pang, Shixing Tang

**Affiliations:** Guangdong Provincial Key Laboratory of Tropical Disease Research, Department of Epidemiology, School of Public Health, Southern Medical University, Guangzhou, China

**Keywords:** SARS-CoV-2 infection, seroprevalence, COVID-19 vaccination, homelessness, meta-analysis

## Abstract

**Objectives:**

SARS-CoV-2 infection and COVID-19 vaccination of homeless people are a serious public health concern during COVID-19 pandemic. We aimed to systematically assess SARS-CoV-2 incidence, seroprevalence, and COVID-19 vaccination coverage in homeless people, which are important to inform resource allocation and policy adjustment for the prevention and control of COVID-19.

**Methods:**

We searched PubMed, Web of Science, and the World Health Organization COVID-19 database for the studies of SARS-CoV-2 incidence, seroprevalence, and COVID-19 vaccination coverage in the homeless population. Subgroup analyses were conducted to pool SARS-CoV-2 incidence and seroprevalence in sheltered homeless, unsheltered homeless, and mixed population, respectively. Potential sources of heterogeneity in the estimates were explored by meta-regression analysis.

**Results:**

Forty-nine eligible studies with a total of 75,402 homeless individuals and 5,000 shelter staff were included in the meta-analysis. The pooled incidence of SARS-CoV-2 infection was 10% (95% CI: 7 to 12%) in the homeless population and 8% (5 to 12%) for shelter staff. In addition, the overall estimated SARS-CoV-2 specific seroprevalence was 19% (8 to 33%) for homeless populations and 22% (3 to 52%) for shelter staff, respectively. Moreover, for the homeless subjects, the pooled incidence was 10% (4 to 23%) for asymptomatic SARS-CoV-2 infections, 6% (1 to 12%) for symptomatic SARS-CoV-2 infections, 3% (1 to 4%) for hospitalization for COVID-19, and 1% (0 to 2%) for severe COVID-19 cases, respectively while no COVID-19-related death was reported. Furthermore, the data derived from 12 included studies involving 225,448 homeless individuals revealed that the pooled proportion of one dose COVID-19 vaccination was 41% (35 to 47%), which was significantly lower than those in the general population.

**Conclusion:**

Our study results indicate that the homeless people remain highly susceptible to SARS-CoV-2 infection, but COVID-19 vaccination coverage was lower than the general population, underscoring the need for prioritizing vaccine deployment and implementing enhanced preventive measures targeting this vulnerable group.

## 1. Introduction

As of March 10, 2023, the severe acute respiratory syndrome coronavirus 2 (SARS-CoV-2) has caused more than 670 million infections and approximately 6.9 million deaths with a mortality of ~1.0% (1). Within less than 12 months since the initial outbreak of SARS-CoV-2 infection in late December 2019 (2), a large amount of vaccines against the coronavirus disease 2019 (COVID-19) had been developed based on several different technologies and platforms, and authorized for use around the world (3). Till now, 70.3% of the world population have been vaccinated with at least one dose of COVID-19 vaccine (4). However, SARS-CoV-2 variants are continuously emerging and spreading across the world. SARS-CoV-2 variants of concern including Alpha, Beta, Gamma, Delta, and Omicron show specific biological feature, such as enhanced resistance to immunity protection induced by COVID-19 vaccine (5–10). In addition, waning protection over time against the infection of SARS-CoV-2 and COVID-19 has been documented (11–14). Therefore, the ongoing pandemic of COVID-19 has not yet subsided. It is necessary to timely monitor and track SARS-CoV-2 circulation especially in the marginalized population such as homeless people who might move or travel easily from place to place, and make the tracking and prevention of SARS-CoV-2 transmission more difficult (15).

Homelessness is recognized as a serious issue and challenge of global concern due to the possible unprecedented outbreaks of COVID-19 among these people (16). In general, homeless people staying in shelters (sheltered homeless), or on the streets and other similar settings (unsheltered homeless) are denoted as homelessness (17). In 2019, there were about 700,000 homeless people on a single night in the European Union while the number increased by 70% in a decade (18). According to the 2021 annual homeless assessment report released by the U.S. Department of housing and urban development, there were 326,126 sheltered homeless people on any given night in January of 2021 in the United States (19). Homeless people usually possess increased susceptibility to infectious disease and mental disorders (20, 21), and show poor adherence to public health recommendations and limited access to testing, vaccine, or medical service (17, 22–24). Therefore, the containment of SARS-CoV-2 transmission among homeless people may be difficult and complicated. Specht et al. (25) proposes to enhance health communication with homeless people by bridging the “digital gap” and mitigate the structural marginalization of them. In order to interrupt the spread of SARS-CoV-2 among this vulnerable group and further from them into the general population, a comprehensive analysis to clarify SARS-CoV-2 incidence, seroprevalence, and COVID-19 vaccination coverage in homeless people is important for planning and deploying health services tailored to them (20).

One meta-analysis reported the pooled prevalence of 2.3% at baseline and 31.6% in the situation of SARS-CoV-2 outbreak among homeless people between January 2020 and October 2020 (26). In addition, another study identified a prevalence of symptomatic COVID-19 infection of 35% in the homeless and a higher rate of vaccine hesitancy than the general population during the first year of the pandemic (27). However, since 2021, the global COVID-19 pandemic has changed including the emergence of more transmissible SARS-CoV-2 Omicron variant, and worldwide massive vaccination (28). Furthermore, quite different COVID-19 vaccination rates have been reported in the homeless population (24, 29–39). In this study, we conducted an updated meta-analysis and systematic review on SARS-CoV-2 incidence, seroprevalence, and COVID-19 vaccination coverage in homeless individuals.

## 2. Methods

### 2.1. Search strategy and selection criteria

We searched PubMed, Web of Science, and the World Health Organization COVID-19 database by using the combinations of terms relating to SARS-CoV-2 infection (2019-nCoV OR SARS-CoV-2 OR COVID-19) and being homelessness (homeless* OR roofless OR shelter*) for studies of SARS-CoV-2 incidence and seroprevalence in the homeless population published from December 1, 2019 to July 31, 2022. We also screened the reference lists of all the eligible primary studies as well as the relevant review articles to identify other related studies. The meta-analysis was conducted following the guidelines of Preferred Reporting Items for Systematic Reviews and Meta-Analyses (PRISMA) (40) (Supplementary Checklist S1). Studies on the COVID-19 vaccination coverage in homeless people were identified through searches PubMed, Web of science, the World Health Organization COVID-19 database up to August 10, 2022 using the following search strategy: ((((SARS-CoV-2) OR (Covid-19)) OR (2019-nCoV)) AND (((homeless*) OR (roofless)) OR (shelter*))) AND (vaccine*).

The included studies met the following criteria: (1) study subjects were homeless people; (2) diagnosis of SARS-CoV-2 infection was based on the specific testing assays, such as nucleic acid amplification tests (NAATs), antigen tests, or serological tests (41) (3) anti-SARS-CoV-2 seropositivity was not the immunological response induced by COVID-19 vaccination; (4) the data to determine SARS-CoV-2 incidence or seroprevalence were available. We excluded the studies or papers if: (1) they were reviews, editorial, opinions, case reports or animal studies; (2) the number of homeless individuals was not reported or could not be obtained from the authors.

### 2.2. Data extraction

Three authors (QS, QL, and YP) independently extracted the following information, i.e., the first author, year of publication, study period, country, study subjects, number of the investigated homeless individuals, gender, age, category of homelessness, diagnostic method/criteria and number of homeless people diagnosed with SARS-CoV-2 infection, number of vaccinated people, number of asymptomatic SARS-CoV-2 infections, number of symptomatic SARS-CoV-2 infections, number of COVID-19-related hospitalization, number of severe COVID-19 cases, and COVID-19-related mortality. The severity of illness was assessed according to the seventh version guideline for the diagnosis and treatment of COVID-19 published by the National Health Commission of China (42) and classified into: (1) a symptomatic carriers present with no clinical symptom but with a positive result of the pathogens tests of SARS-CoV-2 in respiratory tract specimens and so on; (2) mild patients have mild clinical symptoms and no pneumonia on chest imaging; (3) moderate patients have clinical symptoms (i.e., fever and respiratory tract symptoms) and pneumonia on chest imaging. (4) Severe patients who meet any one of the following criteria: respiratory rate ≥30 breaths/min; resting oxygen saturation ≤93%; arterial partial pressure of oxygen (PaO_2_)/oxygen concentration (FiO_2_) ≤300 mmHg; disease progression within 24 to 48 h on chest image. Any disagreement between the three authors was resolved by discussing with the corresponding author YL or ST to reach a consensus.

### 2.3. Quality assessment

The methodological quality of the included studies was assessed using an 11-item checklist which was recommended by Agency for Healthcare Research and Quality (AHRQ). The total score is the sum of the scores for each item, with a score of “yes” giving 1 point, a score of “no” giving −1 point, and a score of “unclear” giving 0 point (Table 1).

**Table 1:** Quality of the included studies.

Study	11-items											Total score
	1	2	3	4	5	6	7	8	9	10	11	
Tobolowsky et al. (43)	Yes	Unclear	Yes	Yes	No	Yes	No	No	Yes	Yes	Unclear	5
Baggett et al. (44)	Yes	Yes	Yes	Yes	No	Yes	Yes	Unclear	No	Yes	Unclear	7
Baggett et al. (45)	Yes	Yes	Yes	Yes	No	Yes	No	No	No	Yes	Unclear	4
O’Shea et al. (46)	Yes	Yes	Yes	Yes	No	Yes	Unclear	No	Yes	Yes	Unclear	7
Mosites et al. (47)	Yes	No	Yes	Yes	No	Yes	No	No	Yes	Yes	Unclear	4
Karb et al. (48)	Yes	Yes	Yes	Yes	No	Yes	Yes	No	No	Yes	Unclear	6
Imbert et al. (49)	Yes	No	Yes	Yes	No	Yes	Yes	No	No	Yes	Unclear	4
Kelly et al. (50)	Yes	Unclear	Yes	Yes	No	Yes	Yes	Yes	Yes	Yes	Unclear	9
Gombita et al. (51)	Yes	No	Yes	Yes	No	Yes	No	No	No	No	Unclear	0
Seballos et al. (52)	Yes	No	Yes	Yes	No	Yes	No	No	No	Yes	Unclear	2
Yoon et al. (53)	Yes	Unclear	Yes	Yes	No	Yes	Yes	Yes	No	Yes	Unclear	7
Wang et al. (54)	Yes	Yes	Yes	Yes	No	Yes	Yes	Yes	No	Yes	Unclear	8
Ralli et al. (55)	Yes	Yes	Yes	Yes	No	Yes	No	No	No	Yes	Unclear	4
Ghinai et al. (56)	Yes	Yes	Yes	Yes	No	Yes	Yes	No	No	Yes	Yes	7
Marquez et al. (57)	Yes	No	Yes	Yes	No	No	No	No	No	No	Unclear	-2
Storgaard et al. (58)	Yes	Yes	Yes	Yes	No	Yes	No	No	Yes	Yes	Unclear	6
Redditt et al. (59)	Yes	Yes	Yes	Yes	No	Yes	Yes	No	No	Yes	Yes	7
Jatt et al. (60)	Yes	Yes	Yes	Yes	No	No	Yes	No	Unclear	Yes	Unclear	5
Baggio et al. (61)	Yes	Yes	Yes	Yes	No	Yes	Yes	No	No	Yes	Unclear	6
Richard et al. (62)	Yes	Yes	Yes	Yes	No	Yes	Yes	Yes	Yes	Yes	Unclear	10
Rogers et al. (63)	Yes	Yes	Yes	Yes	No	Yes	Yes	No	Yes	Yes	Unclear	8
Kiran et al. (64)	Yes	Yes	Yes	Yes	No	Yes	Yes	No	Yes	Yes	Unclear	8
Le Bihan et al. (65)	Yes	Yes	Yes	Yes	No	No	Yes	No	Unclear	Yes	Yes	6
Husain et al. (66)	Yes	Yes	Yes	Yes	No	Yes	Yes	No	No	Yes	Unclear	6
Ly et al. (67)	Yes	No	Yes	Yes	No	Yes	Unclear	No	Unclear	Yes	Unclear	4
Ly et al. (68)	Yes	Yes	Yes	Yes	No	Yes	Unclear	No	Unclear	Yes	Unclear	6
Kiran et al. (69)	Yes	Yes	Yes	Yes	No	Yes	Unclear	No	Unclear	Yes	Unclear	6
Roederer et al. (70)	Yes	Yes	Yes	Yes	No	Yes	Yes	Yes	Yes	Yes	Unclear	10
Roland et al. (71)	Yes	Yes	Yes	Yes	No	Yes	Unclear	No	No	Yes	Unclear	5
Loubiere et al. (72)	Yes	Yes	Yes	Yes	No	Unclear	Yes	Yes	Yes	Yes	Unclear	9
Hsu et al. (73)	Yes	Yes	Yes	Yes	No	Yes	Yes	Yes	Yes	Yes	Yes	11
Keller et al. (74)	Yes	Yes	Yes	Yes	No	Yes	Unclear	No	Unclear	Yes	Unclear	6
do Couto et al. (75)	Yes	Yes	Yes	Yes	No	Yes	Yes	Yes	Unclear	Yes	Unclear	8
Oette et al. (76)	Yes	Unclear	Yes	Yes	No	Yes	Unclear	Unclear	Unclear	Yes	Unclear	5
Ralli et al. (77)	Yes	Yes	Yes	Yes	No	Yes	Unclear	No	Unclear	Yes	Unclear	5
Song et al. (78)	Yes	Yes	Yes	Yes	No	Yes	Unclear	Unclear	Unclear	Unclear	Unclear	5
Lindner et al. (79)	Yes	Yes	Yes	Yes	No	Yes	Yes	Yes	Unclear	Yes	Yes	9
Fini et al. (80)	Yes	Yes	No	Yes	No	Yes	No	No	Unclear	Unclear	Unclear	2
Thomas et al. (81)	Yes	Yes	Yes	Yes	No	Yes	Yes	Yes	Unclear	Yes	Unclear	8
Huggett et al. (82)	Yes	Yes	Yes	Yes	No	Yes	Yes	Yes	Unclear	Yes	Unclear	7
Chang et al. (83)	Yes	Yes	Yes	Yes	No	Yes	Unclear	Unclear	Unclear	Yes	Yes	7
Luong et al. (84)	Yes	Yes	Yes	Yes	No	Yes	Yes	Unclear	No	Yes	Unclear	6
Allibert et al. (85)	Yes	Yes	Yes	Yes	No	Yes	No	Yes	Unclear	Unclear	Unclear	5
Rowan et al. (86)	Yes	Yes	Yes	Yes	No	Yes	Yes	Yes	Unclear	Unclear	Unclear	7
Bojorquez-Chapela et al. (87)	Yes	Yes	Yes	Yes	No	Yes	Yes	Yes	Unclear	Yes	Unclear	8
Aranda-Díaz et al. (88)	Yes	Yes	Yes	Yes	No	Yes	Yes	Yes	No	Yes	Yes	8
Berner et al. (89)	Yes	Yes	Yes	Yes	No	Yes	Unclear	Unclear	Unclear	Yes	Unclear	6
Morrone et al. (90)	Yes	Yes	Yes	Yes	No	Yes	Yes	Unclear	Unclear	Yes	Unclear	7
Eriksen et al. (91)	Yes	Yes	Yes	Yes	No	Yes	Yes	No	Unclear	Yes	No	5

^*^(1) Define the source of information (survey, record review). (2) List inclusion and exclusion criteria for exposed and unexposed subjects (cases and controls or refer to previous publications). (3) Indicate time period used for identifying patients. (4) Indicate whether or not subjects were consecutive if not population-based. (5) Indicate if evaluators of subjective components of study were masked to other aspects of the status of the participants. (6) Describe any assessments undertaken for quality assurance purposes (e.g., test/retest of primary outcome measurements). (7) Explain any patient exclusions from analysis. (8) Describe how confounding was assessed and/or controlled. (9) If applicable, explain how missing data were handled in the analysis. (10) Summarize patient response rates and completeness of data collection. (11) Clarify what follow-up was expected and the percentage of patients for which incomplete data or follow-up was obtained. The total score is the sum of the scores for each item, with a score of “yes” giving 1 point, a score of “no” giving −1 point, and a score of “unclear” giving 0 point. However, for the fifth item, a response of “yes” scores 0 points and “no” scores 1 point.

### 2.4. Statistical analysis

The SARS-CoV-2 incidence or seroprevalence estimated by individual study was transformed with the Freeman–Tukey double arcsine function before pooling the incidence or seroprevalence to decrease the effect of studies with extremely low frequency on the overall estimate (92). Since the asymptotic method produces intervals that may extend below zero, the 95% confidence intervals (CIs) around these estimates were calculated by the Wilson method (93, 94). Moreover, both Cochran’s Q (reported as *χ*^2^ value and *p*-value) and the *I*^2^ statistic were applied to estimate the inter-studies heterogeneity. A *p* < 0.05 from Cochrane’s chi-square (*χ*^2^) test or *I*^2^ statistic value >75% indicated substantial heterogeneity (95, 96). A random effect model was used in the situations with substantial inter-studies heterogeneity; otherwise, a fixed effect model was adapted (95). Publication bias was assessed by using Egger and Begg tests (97, 98). Furthermore, subgroup analyses were conducted to explore the SARS-CoV-2 incidence and seroprevalence according to homelessness category (sheltered, unsheltered, and mixed population). If repeat testing was performed in the given shelter for the homeless, the screening with the largest sample size was included in quantitative synthesis. We have also conducted an additional analysis that compared the incidence of SARS-CoV-2 infection in homeless people with the estimated cumulative incidence of SARS-CoV-2 in the total general population during corresponding period to calculate incidence ratios. Information about the cumulative incidence of SARS-CoV-2 in the total general population by country or region was obtained from Our World in Data.^1^ All the analyses were done by using the Package “meta” in R software (version 4.2.1, R Foundation for Statistical Computing). A two-sided *p* < 0.05 was considered statistically significant.

## 3. Results

### 3.1. Study selection

**Figure 1:**
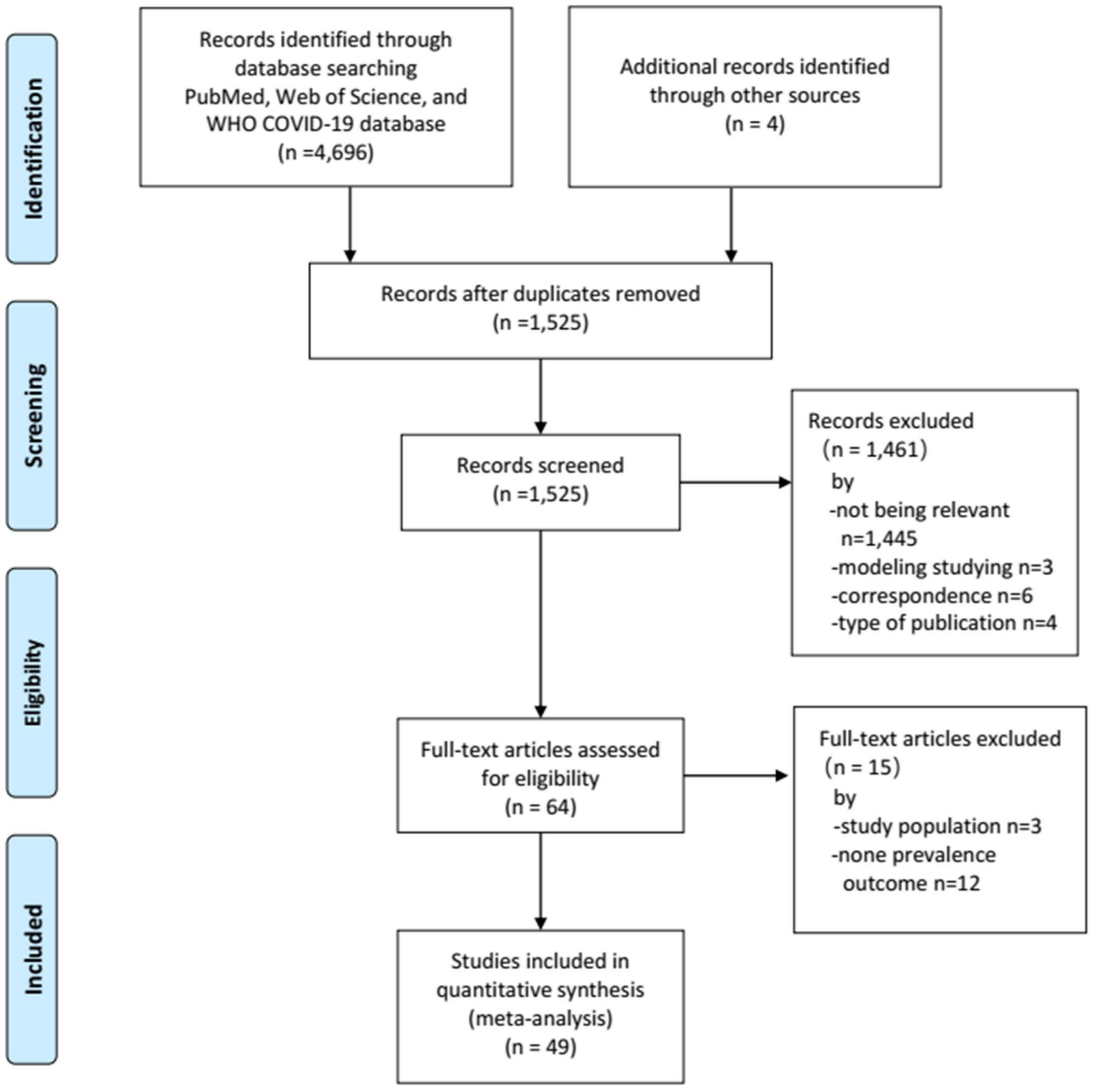
Flow-chart depicting the systematic search conducted to identify eligible studies.

Our literature search yielded a total of 4,696 records, of which 1,230 were retrieved from PubMed, 1,425 from Web of Science, and 2,041 from WHO COVID-19 database. An additional 4 reports were identified from the reference lists of the relevant review articles. After removing the duplicates, 1,525 titles and abstracts were eligible for screening. Of these, 1,461 studies were discarded after reviewing the titles and abstracts. Furthermore, 15 studies were discarded after full-text screening. Finally, 49 studies (43–91) involving 75,402 homeless individuals met the eligibility criteria and were included in the meta-analysis (Figure 1).

### 3.2. Characteristics of the included studies

Out of the 49 included studies (Supplementary Table S1), 20 eligible studies (*N* = 29,513) were conducted in the United States (43–45, 47–50, 52, 53, 56, 57, 60, 63, 73, 74, 82, 83, 86, 88, 89), 7 (*N* = 25,074) in Canada (46, 54, 59, 62, 64, 69, 84), 7 (*N* = 3,957) in France (65–68, 70, 72, 85), 2 (*N* = 4,870) in the United Kingdom (78, 81), 2 (*N* = 757) in Denmark (58, 91), 2 (*N* = 181) in Germany (76, 79), 2 (*N* = 6,641) in Italy (55, 90), and one each from Belgium (*N* = 1,985) (71), Brazil (*N* = 203) (75), Iran (*N* = 234) (80), Mexico (*N* = 481) (87), Slovakia (*N* = 331) (51), Switzerland (*N* = 215) (61) and Vatican (*N* = 960) (77), respectively. The estimated pooled median age of 45.0 (95% CI, 42.9, 47.1) years was reported in 33 articles, and 37 publications reported gender of homeless people whose majority was male.

The majority (83.7%, 41/49) of the included studies was cross-sectional study. Thirty-two studies reported the SARS-CoV-2 incidence or seroprevalence in sheltered homeless and their median sample size was 331 (QTR 51-11,463) (43, 44, 46–51, 53, 54, 56–60, 63, 66–69, 71, 73, 75, 79, 82, 83, 86–89, 91) while 15 studies also simultaneously investigated SARS-CoV-2 incidence or seroprevalence among the shelter staff (*N* = 5,000) (43, 46, 47, 49, 53, 56, 63, 66, 67, 75, 77, 83, 88, 89, 91) (Supplementary Tables S1, S2). Four studies (*N* = 1,351) (53, 65, 80, 86) were conducted in the unsheltered homeless people while 15 (*N* = 31,232) (44, 52, 55, 61, 62, 70, 72, 74, 76–78, 81, 84, 85, 90) in the mixed population comprising sheltered and unsheltered homeless subjects whose SARS-CoV-2 incidence or seroprevalence was not separately reported. For the diagnosis of active SARS-CoV-2 infection in homeless people, 40 studies were based on NAATs alone, 1 study was based on antigen tests alone, and 1 investigation was based on the combination of NAAT and antigen tests. Moreover, the seroprevalence of SARS-CoV-2 was evaluated in the homeless population in 11 surveys (51, 55, 58, 66, 70, 72, 75, 85–87, 91) (Supplementary Table S1).

### 3.3. SARS-CoV-2 incidence and seroprevalence in the homeless population

**Figure 2:**
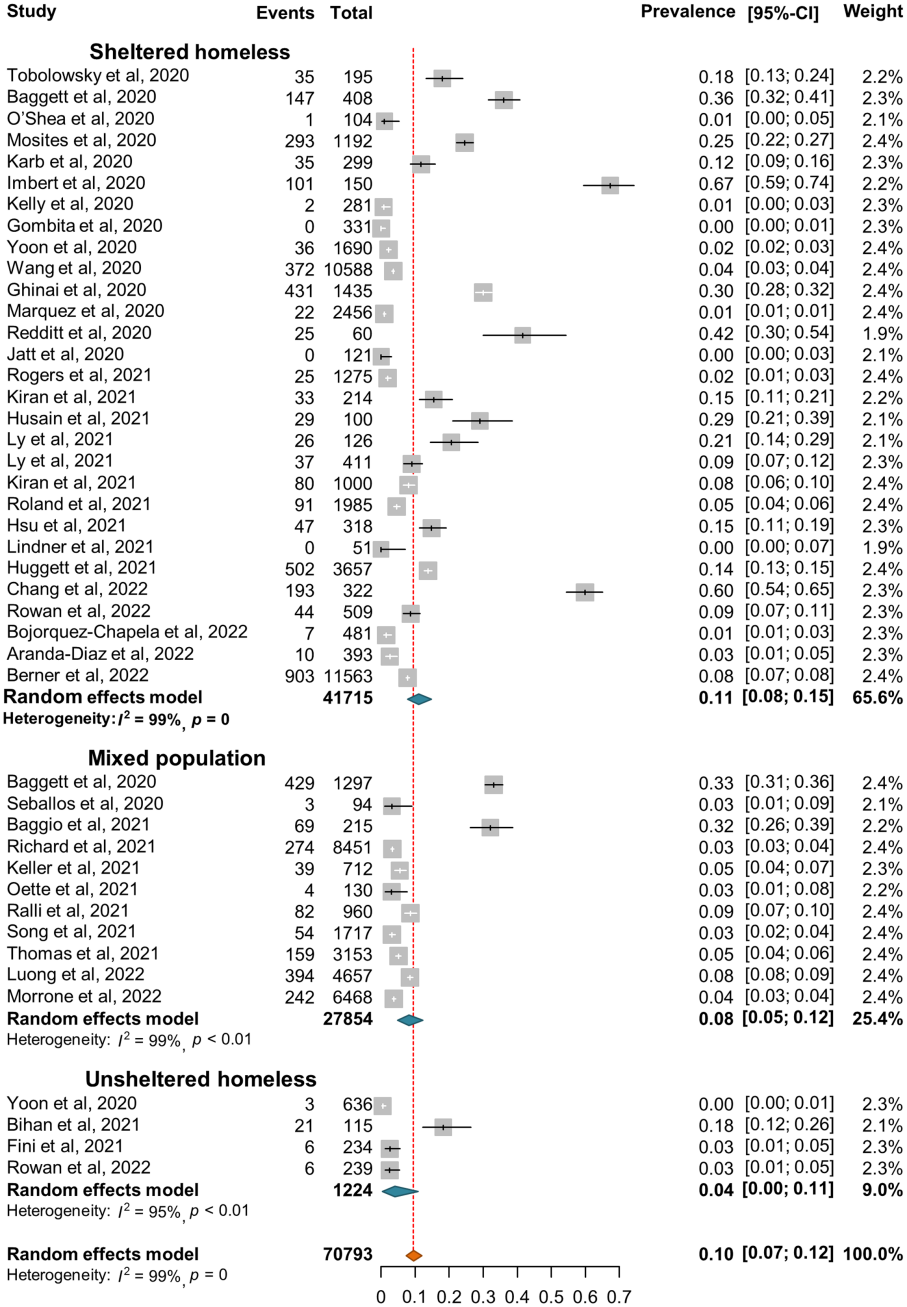
Forest plots of the estimated incidence of SARS-CoV-2 infection in homeless people according to the category of homeless.

**Figure 3:**
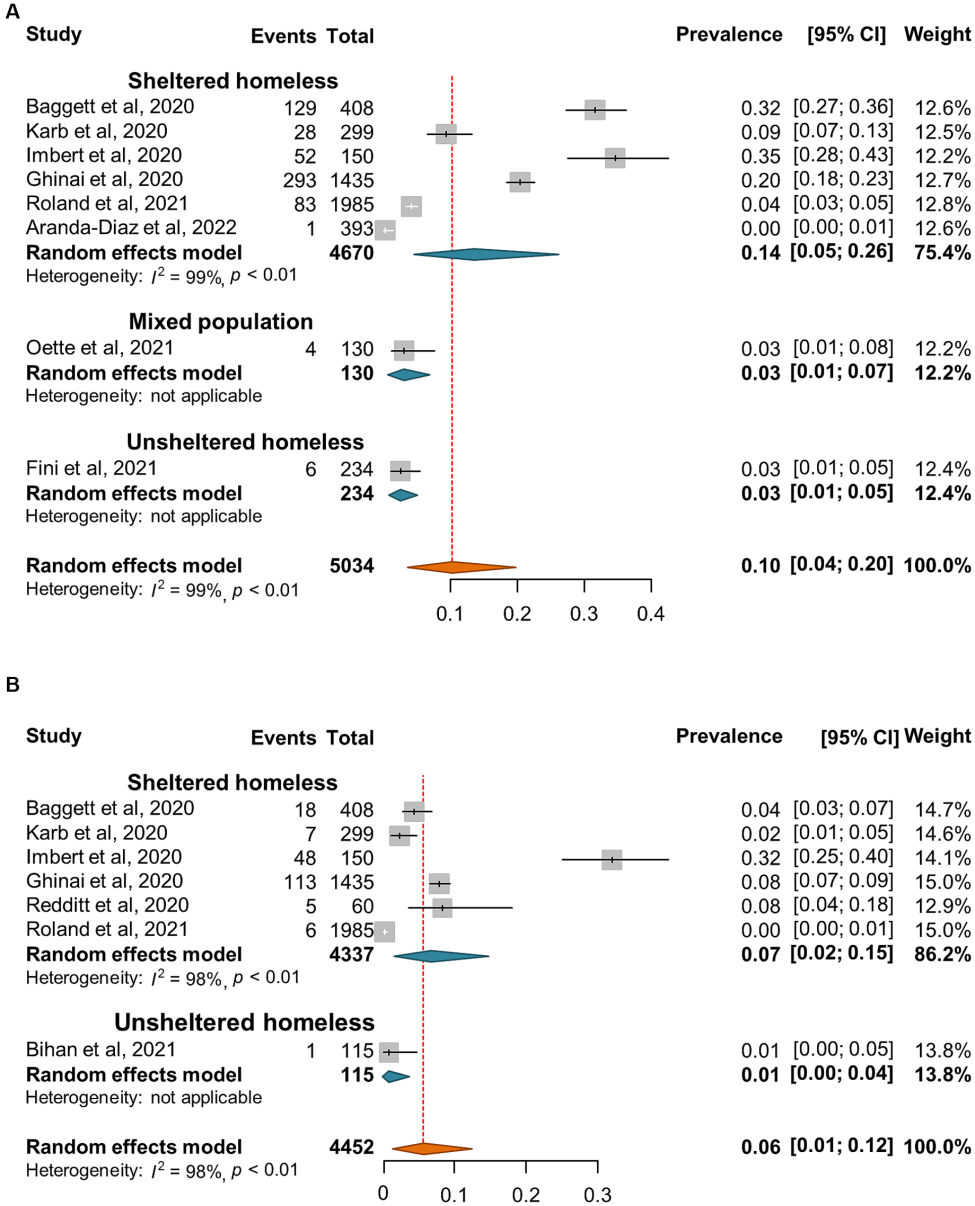
Forest plots of the estimated incidence of asymptomatic SARS-CoV-2 infection **(A)** and symptomatic infection **(B)** in homeless people according to the category of homeless.

**Figure 4:**
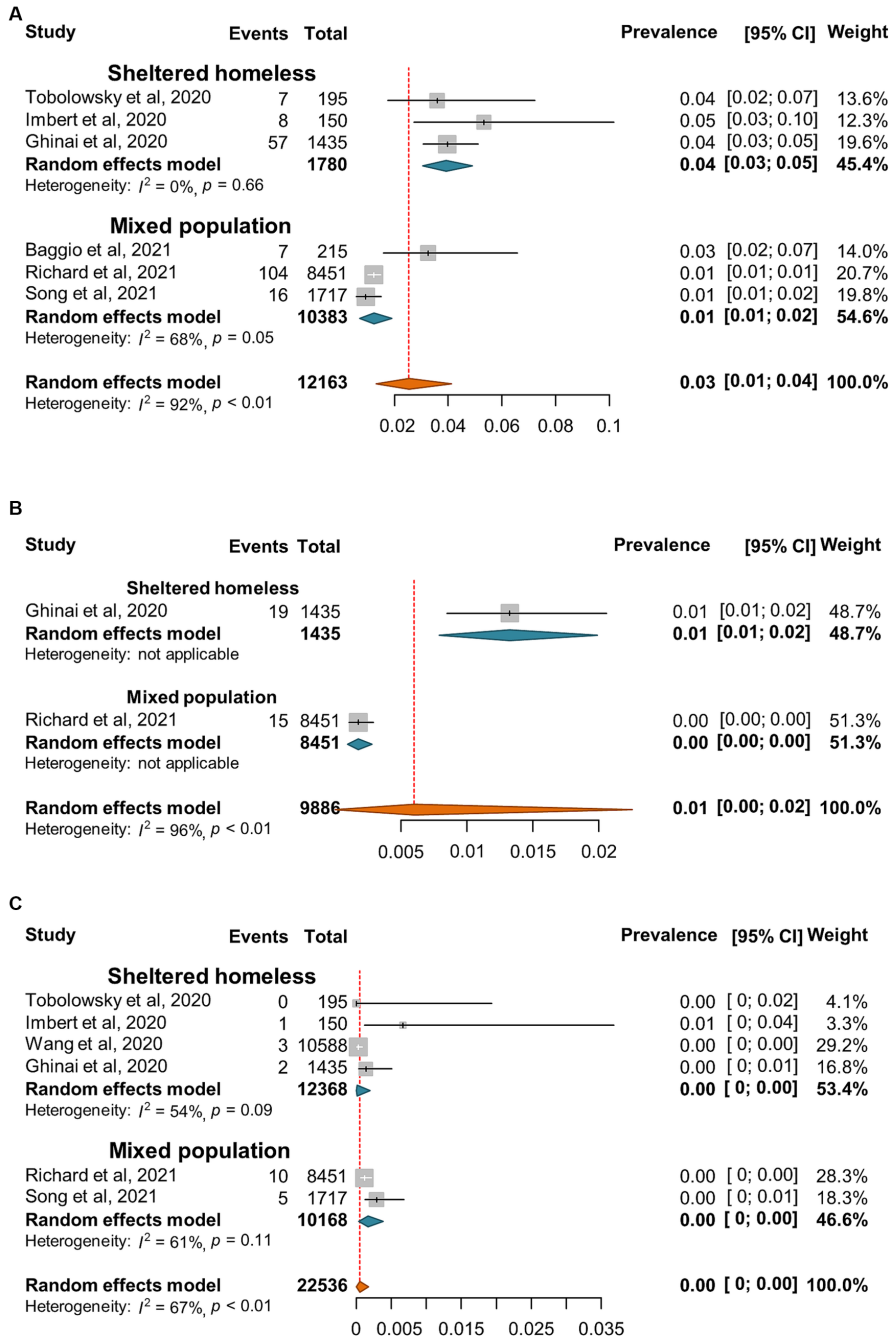
Forest plots of the estimated prevalence of hospitalization **(A)**, sever cases **(B)**, and death **(C)** caused by SARS-CoV-2 infection in homeless people according to the category of homeless.

SARS-CoV-2 incidence ranged from 0 to 67% with very high heterogeneity among the studies (*I*^2^ = 99%, *p* = 0) (Figure 2). The random-effect pooled incidence of SARS-CoV-2 infection was 10% (95% CI, 7, 12%) whereas 11% (8, 15%) for sheltered homeless, 4% (0, 11%) for unsheltered homeless, and 8% (5, 12%) for the mixed population, respectively (Figure 2). Moreover, the random-effect pooled incidence was 10% (4, 23%) for asymptomatic SARS-CoV-2 infections (Figure 3A), 6% (1, 12%) for symptomatic SARS-CoV-2 infections (Figure 3B), 3% (1, 4%) for the COVID-19-related hospitalization (Figure 4A), 1% (0, 2%) for severe COVID-19 (Figure 4B), respectively although no COVID-19-related death was reported (Figure 4C). Of note, the random-effect pooled incidence of SARS-CoV-2 infection remained 10% (8, 12%) with substantial heterogeneity (*I*^2^ = 99%, *p* = 0) when SARS-CoV-2 infection was diagnosed by NAATs alone in homeless people (Supplementary Figure S1).

Interestingly, in 2020, all the studies showed higher SARS-CoV-2 incidence in homeless people than in the general population and the SARS-CoV-2 incidence ratio between homeless people and general population was 1.8–94.6 (Table 2). However, 4 studies in the United Kingdom, Italy and Mexico showed a reversed SARS-CoV-2 incidence ratio, which ranged from 0.5 to 0.8 between homeless people and general population (Table 2).

**Table 2:** Comparison of incidence of SARS-CoV-2 infection between homeless people and general population.

Country	Homeless population			General population				Incidence ratio of homeless vs. general population
	No. of study	Study period	Random-effect pooled incidence (95% CI)	Study period	Total cases	General population	Cumulative incidence (%)	
**Studies data collected in 2020**								
USA	18	2020/01–2020/09	14.3 (8.8 to 21.0)	2020/01–2020/09	7,240,030	332,915,074	2.2	6.6
Canada	7	2020/01–2020/07	7.9 (5.2 to 11.0)	2020/01–2020/07	116,886	38,067,913	0.3	25.7
France	4	2020/03–2020/08	18.3 (9.7 to 28.8)	2020/01–2020/08	320,559	67,422,000	0.5	38.5
Germany	2	2020/05–2020/06	1.4 (0 to 5.6)	2020/01–2020/06	194,259	83,900,471	0.2	6.0
Belgium	1	2020/04–2020/06	4.6 (3.7 to 5.5)	2020/01–2020/06	61,427	11,632,334	0.5	8.7
Iran	1	2020	2.6 (0.9 to 5.1)	2020/01–2020/12	1,225,142	85,028,760	1.4	1.8
Switzerland	1	2020/03–2020/04	32.1 (26.0 to 38.5)	2020/01–2020/04	29,586	8,715,494	0.3	94.6
Slovakia	1	2020/03–2020/06	0 (0 to 0.5)	2020/01–2020/06	1,667	5,449,270	0.0	NA
**Studies data collected from 2020 to 2021**								
UK	2	2020/03–2021/03	4.1 (2.4 to 6.1)	2020/01–2021/03	4,349,834	68,207,114	6.4	0.6
Mexico	1	2020/11–2021/04	1.5 (0.6 to 2.8)	2020/01–2021/04	2,344,755	130,262,220	1.8	0.8
Vatican	1	2020/10–2021/06	8.5 (6.9 to 10.4)	2020/01–2021/06	27	812	3.3	2.6
Italy	1	2020/03–2021/10	3.7 (3.3 to 4.2)	2020/01–2021/10	4,771,965	60,367,471	7.9	0.5

NA, not applicable.

**Figure 5:**
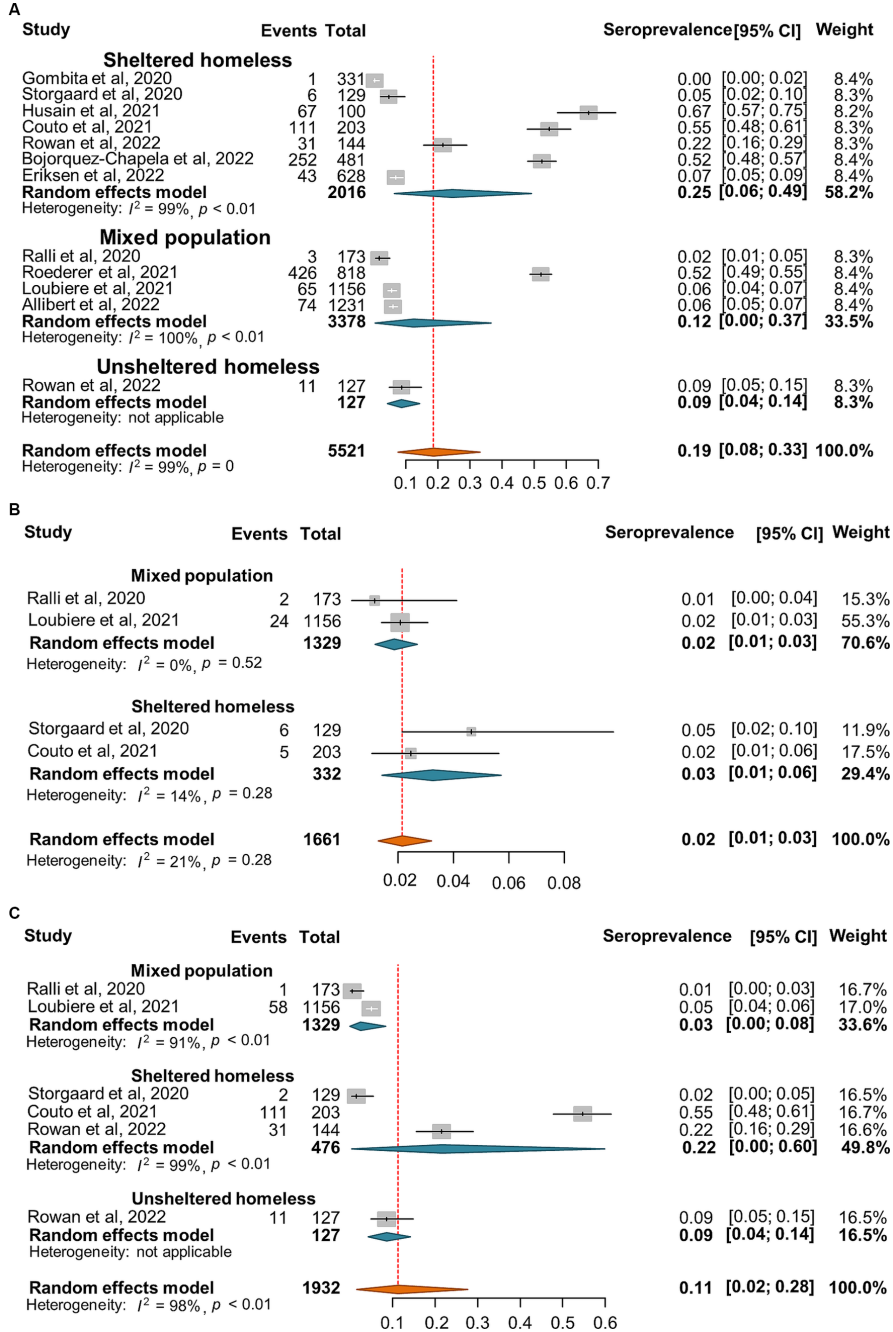
Forest plots of the estimated seroprevalence of anti-SARS-CoV-2 antibodies **(A)**, SARS-CoV-2 specific IgG antibody **(B)**, and SARS-CoV-2 specific IgM antibody **(C)** in homeless people according to the category of homeless.

Furthermore, the seroprevalence of SARS-CoV-2 ranged between 0 and 67% with a random-effect pooled estimate of 19% (8, 33%) and substantial heterogeneity (*I*^2^ = 99%, *p* = 0) in the homeless group (Figure 5A). Moreover, there are 4 and 5 articles reported the number of anti-SARS-CoV-2 IgM and IgG positive subjects, respectively. The random effect pooled seropositivity was 2% (1, 3%) for anti-SARS-CoV-2 IgM, and 11% (2, 28%) for anti-SARS-CoV-2 IgG, respectively (Figures 5B,C).

### 3.4. Factors associated with the SARS-CoV-2 incidence or seroprevalence in the homeless population

Substantial heterogeneity was observed between the primary studies; therefore, we explored the potential sources of variations through multivariable meta-regression analysis. Our results indicated that both the incidence and seroprevalence of SARS-CoV-2 infection were not significantly associated with the factors of study period (2021 vs. 2020), study region (Europe vs. America), study design (non-cross-sectional vs. cross-sectional), category of homelessness (unsheltered vs. sheltered; mixed population vs. sheltered), sample size, and mean/median age (Table 3).

**Table 3:** Multivariable meta-regression analysis for SARS-CoV-2 incidence and seroprevalence in homeless people.

Characteristic	SARS-CoV-2 incidence		Anti-SARS-CoV-2 seroprevalence	
	Meta-regression coefficient [95% CI]	*p*-value	Meta-regression coefficient [95% CI]	*p*-value
**Study period**				
2021 vs. 2020	−0.189 [−0.375 to 0.155]	0.281	0.286 [−1.244 to 1.816]	0.715
**Study region**				
Europe vs. America	−0.069 [−0.290 to 0.152]	0.542	0.156 [−0.687 to 0.999]	0.716
**Study design**				
Non-cross-sectional vs. Cross-sectional	−0.221 [−0.529 to 0.087]	0.16	0.362 [−0.484 to 1.209]	0.402
Category of homeless				
Unsheltered vs. sheltered	−0.179 [−0.466 to 0.109]	0.224	−0.317[−1.265 to 0.630]	0.512
Mixed population vs. sheltered	0.168 [−0.138 to 0.475]	0.282	−0.167 [−1.427 to 1.092]	0.795
Sample size	0	0.185	−0.001 [−0.002 to 0]	0.102
Mean/median age	−0.004 [−0.020 to 0.011]	0.584	−0.018 [−0.109 to 0.073]	0.7

### 3.5. SARS-CoV-2 incidence and seroprevalence among shelter staff

**Figure 6:**
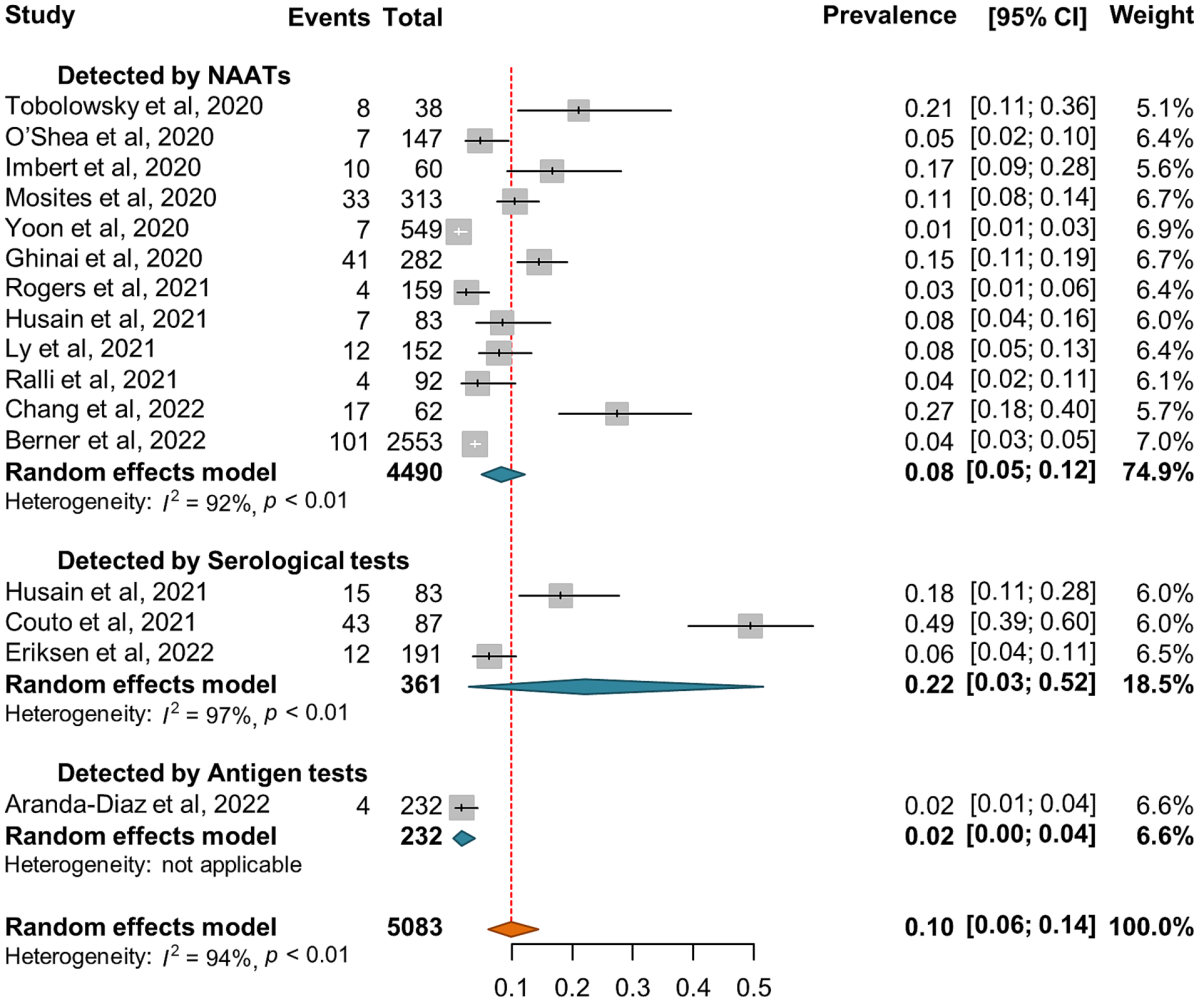
Forest plots of the estimated SARS-CoV-2 incidence and seroprevalence in shelter staff according to the diagnostic method.

Out of the 15 studies that investigated SARS-CoV-2 incidence or seroprevalence among the shelter staff (Supplementary Table S2), there were 12 and 1 investigation diagnosed SARS-CoV-2 infection by NAATs and antigen tests, respectively. The random-effect pooled incidence of SARS-CoV-2 infection was 8% (5, 12%) for diagnosis by NAATs alone and 2% (0, 4%) for antigen tests, respectively (Figure 6). The seroprevalence of SARS-CoV-2 was reported in 3 studies with an estimated pooled seroprevalence of 22% (3, 52%) (Figure 6).

### 3.6. COVID-19 vaccination coverage in the homeless population

**Figure 7:**
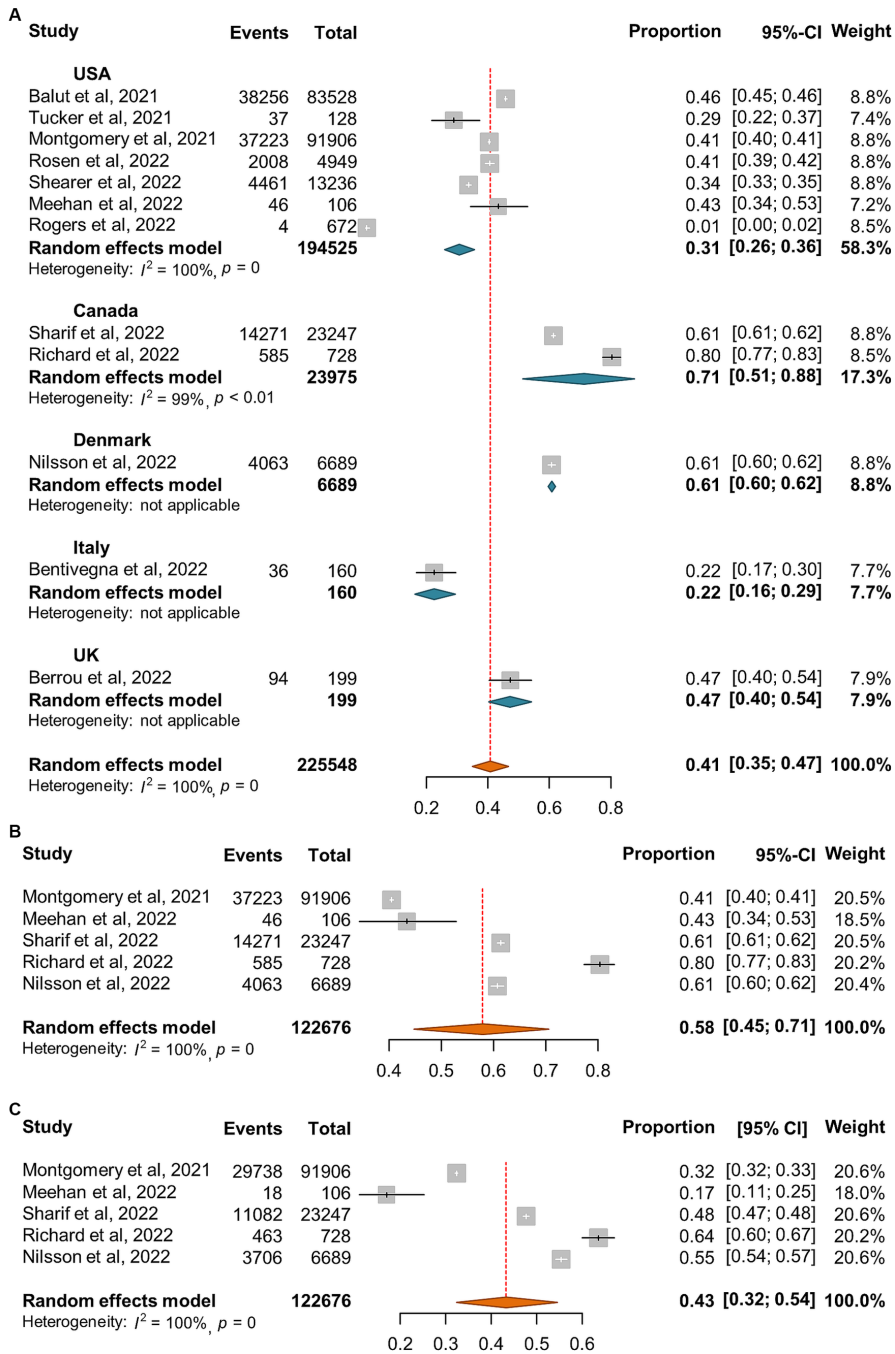
Forest plots of the estimated proportion of vaccinated homeless people. **(A)** One dose vaccination coverage derived from all studies. **(B)** One dose vaccination coverage derived from 5 studies that reported both one dose and two dose vaccination coverage. **(C)** Two dose vaccination coverage derived from 5 studies that reported both one and two dose vaccination coverage.

A total of 12 reports (24, 29–39) involving 225,448 homeless individuals were selected to assess COVID-19 vaccination and the median sample size of the eligible studies was 2,839 (IQR: 106-83,528) (Supplementary Table S3). All the included studies were conducted in North America (7 in United States and 2 in Canada) and Europe (one each from Denmark, Italy, and United Kingdom, respectively). Out of the 12 studies, 5 reported the proportion of homeless people who had received two doses of COVID-19 vaccine (24, 32, 33, 35, 37). Overall, the pooled proportion of homeless people received at least one dose vaccine was 41% (95% CI: 35, 47%, Figure 7A). The results from 5 studies that reported two doses vaccination coverage showed that 58% (45, 71%) and 43% (32, 54%) of homeless people received one dose and two doses vaccine, respectively (Figures 7B,C). In addition, COVID-19 vaccination coverage in the general population was obtained from 9 studies (24, 29–36) or the global database of COVID-19 vaccinations (4) while one study reported COVID-19 vaccination coverage in the general population aged 18–39 years (99). The proportion ratio between homeless people and the general population was 0.04–2.57 for one dose vaccination and 0.58–1.88 for two doses vaccination, respectively (Table 4).

**Table 4:** Comparison of proportion of COVID-19 vaccination between homeless people and general population during 2020 and 2021.

Study	Country	Study period	Proportion of one dose vaccination			Proportion of two doses vaccination		
			Homeless people	General population	Proportion ratio	Homeless people	General population	Proportion ratio
Balut et al. (29)	USA	2020/12–2021/08	45.8%	64.3%	0.71 (0.59 to 0.82)	NA	NA	NA
Tucker et al. (39)	USA	2021/03–2021/10	28.9%	34.0%	0.85 (0.70 to 0.95)	NA	NA	NA
Rosen et al. (34)	USA	2021/05–2021/11	40.6%	73.0%	0.56 (0.44 to 0.68)	NA	NA	NA
Shearer et al. (36)	USA	2021	33.7%	64.0%	0.53 (0.40 to 0.66)	NA	NA	NA
Rogers et al. (38)	USA	2020/11–2021/02	0.6%	16.9%	0.04 (0 to 0.18)	NA	NA	NA
Berrou et al. (31)	UK	2020/12–2021/05	47.3%	84.4%	0.56 (0.45 to 0.67)	NA	NA	NA
Bentivegna et al. (30)	Italy	2021/06–2021/09	22.5%	79.1%	0.28 (0.19 to 0.40)	NA	NA	NA
Montgomery et al. (24)	USA	2020/12–2021/08	40.5%	60.7%	0.67 (0.54 to 0.79)	32.4%	53.8%	0.60 (0.45 to 0.72)
Meehan et al. (32)	USA	2021.02	43.4%	16.9%	2.57 (1.80 to 4.00)	17.0%	9.0%	1.88 (1.30 to 3.60)
Sharif et al. (35)	Canada	2020/12–2021/09	61.4%	86.6%	0.71 (0.60 to 0.79)	47.7%	81.6%	0.58 (0.47 to 0.69)
Richard et al. (37)	Canada	2021/06–2021/09	80.4%	84.3%	0.95 (0.88 to 0.99)	46.3%	70.9%	0.65 (0.53 to 0.76)
Nilsson et al. (33)	Denmark	2020/12–2021/10	60.7%	86.7%	0.70 (0.59 to 0.79)	55.4%	85.1%	0.65 (0.54 to 0.75)

NA, not available.

### 3.7. Publication bias

Potential publication bias was assessed by Egger and Begg tests. Overall, no evidence of significant publication bias was obtained for the surveys that investigated SARS-CoV-2 incidence (Egger test, *p* = 0.065; Begg test, *p* = 0.093) and seroprevalence (Egger test, *p* = 0.585; Begg test, *p* = 0.411) among homeless people. In addition, the result of Egger test (*p* = 0.036) and Begg test (*p* = 0.131) suggested that the possibility of publication bias was less in the estimated incidence of SARS-CoV-2 infection in shelter staff. Moreover, no significant publication bias was observed for the studies on COVID-19 vaccination coverage of homeless people (Egger test, *p* = 0.963; Begg test, *p* = 0.784).

## 4. Discussion

People experiencing homelessness (PEH) are susceptible to infections including SARS-CoV-2 infection because of inadequate access to safe housing, personal protective equipment, vaccine or healthcare and fragile psychiatric conditions due to social marginalization (100). The current meta-analysis confirmed relatively high risk of SARS-CoV-2 infection in homeless people since the pooled incidence and seroprevalence of SARS-CoV-2 infection was 10 and 19% for the homeless population, higher than in the general population (Table 2). Moreover, the global pooled SARS-CoV-2 specific seroprevalence was less than 10% in the general population (101, 102); however, our estimated seroprevalence was 19% for homeless populations and 22% for shelter staff. Therefore, both homeless people and shelter staff are at higher risk of SARS-CoV-2 infection than the general population. Interestingly, our results indicated that the random-effects pooled incidence of SARS-CoV-2 infection was 11% for the sheltered homeless, 4% for the unsheltered homeless, and 8% for the mixed population, respectively (Figure 1), suggesting that sheltered homeless people may be at greater risk of infecting SARS-CoV-2 probably because the sheltered homeless people are often crowded, and difficult to keep social distance. It is worth mentioning that very few of deaths of homeless population caused by COVID-19 were estimated in the current study (Figure 4C). It was hypothesized that implementation of preventive and control interventions for the pandemic, e.g., lockdown and increased infection control, might have reduced large numbers of deaths in homeless people during the pandemic (103).

Of note, no significant difference of SARS-CoV-2 incidence and seroprevalence was observed in our study between shelter staff (Figure 6) and sheltered homeless people (Supplementary Figure S1; Figure 5). Rao et al. (104) reported that 24% of the shelter staff did not use masks all of the time during the interactions with the homeless while 43% of shelter staff had not received training on cleaning surfaces for SARS-CoV-2, which may put shelter staff at increased risk of exposure to SARS-CoV-2 while very limited hygiene resources in the homeless shelter and poor protection awareness for both homeless people and shelter staff may aggravate the mutual transmission of SARS-CoV-2 (104, 105). In addition, some former homeless residents are employed as shelter staff, which may have narrowed the difference between the two groups (104). Furthermore, most of shelter worker have experienced a decline in their mental health and increase of depression, anxiety, stress and fatigue during the COVID-19 pandemic (106). Similarly, homeless people are susceptible to mental disorders which in turn may increase their vulnerability to the infection of SARS-CoV-2 (21).

Incidence ratios suggested that active SARS-CoV-2 infection is at least about 6.6 times more common in homeless people than in total populations in the United States during 2020 (Table 2). However, when the cumulative incidence of general population in the same country during the same study period was used as reference, the incidence ratio might be underestimated. Moreover, during 2020 the SARS-CoV-2 incidence of homeless people is higher than that of general population across various countries or region, whereas the analysis of studies data involving 2021 showed different results (Table 2). The higher SARS-CoV-2 incidence of general population than homeless people in 2021 may be attributed to loosen travel and gathering restriction (107).

Our results confirmed the lower COVID-19 vaccination coverage rate in homeless people than the general population (Table 4) although some contradictive results were reported by Meehan et al. (32) in Detroit (Table 4) (4). However, another study conducted by Rogers and colleagues found that during November 2020 and February 2021, only 0.6% sheltered homeless people in Washington had been vaccinated (38). In addition, 88.3% of the investigated homeless people were Black or African American in Meehan’s report (32) while 37.4% in Rogers’s one (38). However, according to one meta-analysis of COVID-19 vaccine attitudes in the United States, Black American showed the lowest vaccine acceptance (108). Other studies also showed that the proportion of vaccinated Black American was lower than that of White or Hispanic American (36, 109). Therefore, the lower vaccination rate among the homeless may be partially attributed to reduced willingness to be vaccinated (110). Moreover, our results indicated that one dose vaccination was higher than two doses vaccination (58% vs. 43%, Figures 7B,C).

There are some limitations in the current study. First, since only 5 included studies collected data in 2021, and almost none of them involved vaccinated homeless populations; therefore, we were unable to compare the incidence of SARS-COV-2 infection among homeless people between the pre-vaccination period versus post-vaccination period. Moreover, the number of SARS-COV-2 Delta variant-infected cases reached peak in August 2021 (111) and the Omicron variant outcompeted other counterparts and predominantly circulates globally since its emergence around the end of 2021. However, we did not perform a comparison of the SARS-CoV-2 incidence in homelessness between different pandemic periods that experienced the shifting of predominant variants from Delta to Omicron due to the lack of available data. Furthermore, due to the distribution of latent period, i.e., the time interval between infection (dates of exposure) and becoming infectious (dates of first positive PCR test), the SARS-CoV-2 incidence diagnosed by NAATs might be underestimated. Similarly, the incidence of symptomatic infection would also be underestimated because of the existence of incubation period (the time interval from infection through symptom onset). Given that, further research is needed to better understand the incidence and risk factors of SARS-CoV-2 infection in the homeless populations.

Our study has important implications for public health. Firstly, it highlights the need for targeted interventions to address the high incidence and low vaccination rates among homeless individuals. This could involve strategies such as increasing access to testing, vaccines, healthcare services, as well as personal protective equipment to reduce transmission. Secondly, the study underscores the necessity of addressing health disparities in vulnerable populations and promoting health equity and social justice, particularly during public health crises such as the COVID-19 pandemic. Overall, the study provides important information that will be useful in developing effective policies to protect homeless individuals and the broader public from COVID-19.

## 5. Conclusion

The current study suggests that the homeless people remain highly susceptible to SARS-CoV-2 infection, but their COVID-19 vaccination coverage is lower than general population. These results underscore the need for prioritizing vaccine deployment and implementing enhanced preventive measures targeting this vulnerable group.

## Data availability statement

The original contributions presented in the study are included in the article/Supplementary material, further inquiries can be directed to the corresponding authors.

## Author contributions

YL conceived the study, analyzed, interpreted the data, and drafted the manuscript. QS, QL, and YP performed the literature searches, study selection, and data extraction. YL, QS, QL, YP, and ST revised the manuscript. All authors contributed to the article and approved the submitted version.

## Footnotes

^1^https://ourworldindata.org/coronavirus
