# Supplementary material for: SARS-CoV-2 incidence, seroprevalence, and COVID-19 vaccination coverage in the homeless population: a systematic review and meta-analysis
Source: Front Public Health. 2023 Oct 10;11:1044788. doi: 10.3389/fpubh.2023.1044788 (PMC10600393; doi:10.3389/fpubh.2023.1044788)
Supplement: Supplementary file 1 [file Data_Sheet_1.PDF]

Supplementary Table S1. Details of studies on SARS-CoV-2 incidence and seroprevalence in the homeless population

Study	Country	Study period	Study design	Sample size	Category of Homeless*	Sex	Mean age (years)	Diagnostic Method*	Number of infections†
**Tobolowsky et al, 2020 [59]**	Seattle and King County, USA	2020/03 ~2020/04	Cross-section	195	Sheltered homeless	Male: 95.9% Female: 4.1%	NA	NAATs	35 infections: 7 hospitalized, and 0 death
**Baggett et al, 2020 [47]**	Boston, USA	2020/04	Cross-section	408	Sheltered homeless	Male: 71.6% Female: 28.2%	51.6 (SD, 12.8)	NAATs	147 infections: 129 asymptomatic and 18 symptomatic
**Baggett et al, 2020 [48]**	Boston, USA	2020/03 ~2020/04	Cross-section	1297	Mixed population	NA	NA	NAATs	429 infections
**O'Shea et al, 2020 [75]**	Hamilton, Canada	2020/03 ~2020/04	Cross-section	104	Sheltered homeless	NA	NA	NAATs	1 infection
**Mosites et al, 2020 [53]**	Seattle, Boston, San Francisco, Atlanta	2020/03 ~2020/04	Cross-section	1192	Sheltered homeless	NA	NA	NAATs	293 infections
**Karb et al, 2020 [52]**	Rhode Island, USA	2020.04	Cross-section	299	Sheltered homeless	Male: 80% Female: 20%	47.9 (Range, 18-85)	NAATs	35 infections: 28 asymptomatic and 7 symptomatic
**Imbert et al, 2020 [67]**	San Francisco, USA	2020/03 ~2020/04	Cross-section	150	Sheltered homeless	Male: 84% Female: 16%	Median 54 (Range, 22-77)	NAATs	101 infections: 52 asymptomatic, 48 symptomatic,8 hospitalized and 1 death
**Kelly et al, 2020 [68]**	Michigan, USA	2020/03 ~2020/04	Cross-section	281	Sheltered homeless	NA	NA	NAATs	2 infections
**Gombita et**	Slovakia	2020/03	Cross-section	331	Sheltered	NA	All > 60	NAATs	0

**al, 2020 [50]**		~2020/06			homeless			Serological tests	1 seropositive for antibody test
**Seballos et al, 2020 [56]**	Ohio and Florida, USA	2020/03 ~2020/04	Retrospective cohort study	94	Mixed population	NA	NA	NAATs	3 infections
**Yoon et al, 2020 [84]**	Atlanta, USA	2020/04 ~2020/05	Cross-section	1690	Sheltered homeless	Male: 66.5% Female: 29.8%	44.1	NAATs	36 infections
				636	Unsheltered homeless	Male: 85.1% Female: 14.0%	51.2	NAATs	3 infections
**Wang et al, 2020 [60]**	the Greater Toronto Area, Canada	2020/01 ~2020/05	Prospective cohort study	10588	Sheltered homeless	NA	NA	NAATs	372 infections: 3 deaths
**Ralli et al, 2020 [54]**	Rome, Italy	2020/04 ~2020/07	Cross-section	173	Mixed population	Male: 70.5% Female: 29.5%	45.6 (Range, 10-80)	Serological tests	3 Seropositivity: 2 Seropositive for IgM and 1 Seropositive for IgG
**Ghinai et al, 2020 [49]**	Chicago, USA	2020/03 ~2020/05	Cross-section	1435	Sheltered homeless	Male: 72% Female: 28%	Median 52 (IQR, 39-60)	NAATs	431 infections: 293 asymptomatic, 113 symptomatic, 57 hospitalized, 19 ICU and 2 deaths
**Marquez et al, 2020 [73]**	San Diego, USA	2020/04 ~2020/08	Cross-section	2456	Sheltered homeless	Male: 73.3% Female: 26.7%	49.7 (SD, 13.0)	NAATs	22 infections
**Storgaard et al, 2020 [57]**	Aarhus, Denmark	2020/06	Cross-section	129	Sheltered homeless	Male: 78% Female: 22.0%	Median 53 (95%CI, 42-61)	Serological tests	6 Seropositivity: 6 Seropositive for IgM and less than 3 Seropositive for IgG
**Redditt et al, 2020 [55]**	Toronto, Canada	2020/04	Cross-section	60	Sheltered homeless	Male: 80.0% Female: 20.0%	36.0 (SD, 10.0)	NAATs	25 infections: 5 symptomatic
**Jatt et al, 2020 [51]**	Los Angeles, USA	2020/03 ~2020/04	Cross-section	121	Sheltered homeless	Male :100.0%	52.5 (SD, 13.6)	NAATs	0

**Baggio et al, 2021 [61]**	Geneva, Switzerland	2020/03 ~2020/04	Cross-section	215	Mixed population	Male: 48.4% Female: 51.6%	39.8 (SD, 12.7)	NAATs	69 infections: 7 hospitalized
**Richard et al, 2021 [77]**	Ontario, Canada	2020/01 ~2020/07	Retrospective cohort study	8451	Mixed population	Male: 68% Female: 32%	Median 38 (IQR, 28-52)	NAATs	274 infections: 104 hospitalized, 15 severe cases, and 10 deaths
**Rogers et al, 2021 [79]**	Washington, USA	2020/01 ~2020/04	Cross-section	1275	Sheltered homeless	NA	NA	NAATs	25 infections
**Kiran et al, 2021 [91]**	Toronto, Canada	2020/03 ~2020/04	Cross-section	214	Sheltered homeless	Male: 75.7% Female: 24.3%	Median 40.3 (IQR, 31.0-55.5)	NAATs	33 infections
**Bihan et al, 2021 [70]**	Montpellier, France	2020/04 ~2020/05	Cross-section	115	Unsheltered homeless	NA	30	NAATs	21 infections: 1 symptomatic
**Husain et al, 2021 [66]**	Paris, France	2020/03 ~2020/08	Cross-section	100	Sheltered homeless	Male: 65% Female: 35%	Median 54 (IQR, 37-64)	NAATs and Serological tests	72 infections (29 PCR positive and 67 seropositive): 43 symptomatic, 17 hospitalized, and 4 deaths
**Ly et al, 2021 [83]**	Marseille, France	2020/03 ~2020/07	Cross-section	126	Sheltered homeless	Male: 100% Female: 0%	46.2 (SD, 16.0)	NAATs	26 infections
**Ly et al, 2021 [82]**	Marseille, France	2020/03 ~2020/04	Cross-section	411	Sheltered homeless	Male: 89.8% Female: 10.2%	40.4 (SD, 15.6)	NAATs	37 infections
**Kiran et al, 2021 [69]**	Toronto, Canada	2020/04 ~2020/07	Cross-section	1000	Sheltered homeless	Male: 81.7% Female: 18.3%	45.8 (SD, 16.3)	NAATs	80 infections
**Roederer et al, 2021 [78]**	Paris, France	2020/06 ~2020/07	Cross-section	818	Mixed population	Male: 80% Female: 20%	39	Serological tests	426 Seropositivity
**Roland et al, 2021 [80]**	Brussels, Belgium	2020/04 ~2020/06	Cross-section	1985	Sheltered homeless	Male: 67.7% Female: 32.3%	41.9 (SD, 14.4)	NAATs	91 infections: 83 asymptomatic, and 6 symptomatic
**Loubiere et**	Marseille,	2020/06	Cross-section	1156	Mixed	Male: 71.3%	40.2 (SD, 14.3)	Serological	65 Seropositivity: 24

**al, 2021 [72]**	France	~2020/08			population	Female: 28.7%		tests	Seropositive for IgM, 58 Seropositive for IgG, and 17 Seropositive for both IgM and IgG
**Hsu et al, 2021 [64]**	Quincy and Brockton, USA	2020/03 ~2020/05	Prospective cohort study	318	Sheltered homeless	Male: 70.9% Female: 29.1%	50.6 (SD, 11.9)	NAATs	47 infections
**Keller et al, 2021 [90]**	Louisville, USA	2019/03 ~2020/12	Retrospective study	712	Mixed population	Male: 66.6% Female: 33.4%	43.6 (SD, 16.4)	NAATs and Antigen tests	39 infections
**Couto et al, 2021 [62]**	São Paulo, Brazil	2020/08	Cross-section	203	Sheltered homeless	Male: 89.1% Female: 9.9%	NA	Serological tests	111 Seropositivity: 5 Seropositive for IgM, 111 Seropositive for IgG， 5 Seropositive for both IgM and IgG
**Oette et al, 2021 [74]**	Cologne, Germany	2020/05	Cross-section	130	Mixed population	Male: 90.8% Female: 9.2%	NA	NAATs	4 asymptomatic infections
**Ralli et al, 2021 [76]**	Vatican	2020/10 ~2021/06	Cross-section	960	Mixed population	NA	NA	NAATs	82 infections
**Song et al, 2021 [81]**	Wales, UK	2020	Cross-section	1717	Mixed population	Male: 69.2% Female: 30.8%	NA	NAATs	54 infections: 16 hospitalized, and 5 deaths
**Lindner et al, 2021 [71]**	Berlin, Germany	2020/07	Prospective cohort study	51	Sheltered homeless	Male: 79.6% Female: 20.4%	Median 47 (IQR, 35-54)	NAATs	0
**Fini et al, 2021 [63]**	Bandar Abbas, Iran	NA	Cross-section	234	Unsheltered homeless	Male: 100% Female: 0%	Median 39 (Range, 19-60)	NAATs	6 asymptomatic infections
**Thomas et al, 2021 [58]**	Wales, UK	2020/03 ~2021/03	Cross-section	3153	Mixed population	Male: 67.9% Female: 32.1%	38.5 (SD, 14.0)	NAATs	159 infections
**Huggett et al, 2021 [65]**	Chicago, USA	2020/04 ~2020/09	Retrospective cohort study	3657	Sheltered homeless	NA	NA	NAATs	502 infections
**Chang et**	Chicago, USA	2020/03	Cross-section	322	Sheltered	NA	NA	NAATs	193 infections

**al, 2022 [88]**		~2020/05			homeless				
**Luong et al, 2022 [92]**	Toronto, Canada	2020/04 ~2020/07	Cross-section	4657	Mixed population	NA	NA	NAATs	394 infections
**Allibert et al, 2022 [85]**	Marseille, France	2020/06 ~2020/07	Prospective cohort study	1231	Mixed population	Male: 70.29% Female: 29.71%	40.06 （SD,0.40）	Serological tests	74 infections
**Rowan et al, 2022 [94]**	Denver, USA	2020/07 ~2020/08	Cross-section	509	Sheltered homeless	Male: 86.5% Female: 13.5%	Median 48 (IQR, 38-56)	NAATs	44 infections
				144				Serological tests	31 Seropositive for IgG
				239	Unsheltered homeless	Male: 76.7% Female: 23.3%	Median 41 (IQR, 32-51)	NAATs	6 infections
				127				Serological tests	11 Seropositive for IgG
**Bojorquez-Chapela et al, 2022 [87]**	Tijuana, Mexico	2020/11 ~2021/04	Cross-section	481	Sheltered homeless	Male: 44.7% Female: 55.3%	33.2 (SD, 10.7)	NAATs	7 infections
								Serological tests	252 Seropositive for IgM or IgG
**Aranda-Diaz et al, 2022 [86]**	San Francisco, USA	2021/01 ~2021/02	Cross-section	393	Sheltered homeless	Male: 67.0% Female: 30.3%	45.8（95%CI, 44.5–47.1）	Antigen tests	10 infections: 1 asymptomatic
**Berner et al, 2022 [95]**	USA	2020/03 ~2020/04	Cross-section	11563	Sheltered homeless	NA	NA	NAATs	903 infections
**Morrone et al, 2022 [93]**	Rome, Italy	2020/03 ~2021/10	Cross-section	6468	Mixed population	Male: 55.9% Female: 43.9%	Median 40 (Range, 1-85)	NAATs	242 infections
**Eriksen et al, 2022 [89]**	Denmark	2020/11	Cross-section	628	Sheltered homeless	Male: 64.5% Female: 35.5%	Median 50.8 (IQR, 40.9-59.1)	Serological tests	43 Seropositive for IgM or IgG

Abbreviation: NA: Not available; **NAATs**: Nucleic acid amplification tests.

*Mixed population means investigated subjects including both sheltered and unsheltered homeless.

†Asymptomatic carriers present with no clinical symptom but with a positive result of the pathogens tests of SARS-CoV-2 in respiratory tract specimens and so on; Mild patients have mild clinical symptoms and no pneumonia on chest imaging; Moderate patients have clinical symptoms (i.e. fever and respiratory tract symptoms) and pneumonia on chest imaging. Severe patients who meet any one of the following criteria: respiratory rate ≥30 breaths/minute; resting oxygen saturation ≤93%; arterial partial pressure of oxygen (PaO2)/oxygen concentration (FiO2) ≤300mmHg; disease progression within 24 to 48 hours on chest image.

Supplementary Table S2. Details of studies on SARS-CoV-2 incidence and seroprevalence among shelter staff

Study	Country	Study period	Study design	Sample size	Sex	Mean age (years)	Diagnostic Method*	Number of infections†
**Tobolowsky et al, 2020 [59]**	Seattle and King County, USA	2020/03 ~2020/04	Cross-section	38	NA	NA	NAATs	8 infections
**O'Shea et al, 2020 [75]**	Hamilton, Canada	2020/03 ~2020/04	Cross-section	147	NA	NA	NAATs	7 infections
**Imbert et al, 2020 [67]**	San Francisco, USA	2020/03 ~2020/04	Cross-section	60	NA	NA	NAATs	10 infections: 2 asymptomatic, and 5 symptomatic
**Mosites et al, 2020 [53]**	Seattle, Boston, San Francisco, Atlanta	2020/03 ~2020/04	Cross-section	313	NA	NA	NAATs	33 infections
**Yoon et al, 2020 [84]**	Atlanta, USA	2020/04 ~2020/05	Cross-section	549	Male: 55.2% Female: 44.1%	49.1	NAATs	7 infections
**Ghinai et al, 2020 [49]**	Chicago, USA	2020/03 ~2020/05	Cross-section	282	NA	NA	NAATs	41 infections
**Rogers et al, 2021 [79]**	Washington, USA	2020/01 ~2020/04	Cross-section	159	NA	NA	NAATs	4 infections
**Husain et al, 2021 [66]**	Paris, France	2020/03 ~2020/08	Cross-section	83	Male: 33.7% Female: 66.3%	Median 43 (IQR, 32-54)	NAATs and Serological tests	17 infections (7 PCR positive and 15 seropositive): 16 symptomatic
**Ly et al, 2021 [82]**	Marseille, France	2020/03 ~2020/04	Cross-section	152	Male: 55.9% Female: 44.1%	41.9 (SD, 11.1)	NAATs	12 infections
**Couto et al, 2021 [62]**	São Paulo, Brazil	2020/08	Cross-section	87	Male: 50.6% Female: 49.4%	NA	Serological tests	43 Seropositivity: 5 Seropositive for IgM,

								41 Seropositive for IgG, 3 Seropositive for both IgM and IgG
**Ralli et al, 2021 [76]**	Vatican	2020/10 ~2021/06	Cross-section	92	NA	NA	NAATs	4 infections
**Chang et al, 2022 [88]**	Chicago, USA	2020/03 ~2020/05	Cross-section	62	NA	NA	NAATs	17 infections
**Aranda-Diaz et al, 2022 [86]**	San Francisco, USA	2021/01 ~2021/02	Cross-section	232	Male: 41.4% Female: 56.0%	44.9 (95%CI, 43.1–46.7)	Antigen tests	4 infections: 2 symptomatic
**Berner et al, 2022 [95]**	USA	2020/03 ~2020/04	Cross-section	2553	NA	NA	NAATs	101 infections
**Eriksen et al, 2022 [89]**	Denmark	2020/11	Cross-section	191	Male: 25.5% Female: 74.5%	Median 46.6 (IQR, 36.1-55.0)	Serological tests	12 Seropositive for IgM or IgG

Abbreviation: NA: Not available; **NAATs**: Nucleic acid amplification tests.

*Mixed population means investigated subjects including both sheltered and unsheltered homeless.

†Asymptomatic carriers present with no clinical symptom but with a positive result of the pathogens tests of SARS-CoV-2 in respiratory tract specimens and so on; Mild patients have mild clinical symptoms and no pneumonia on chest imaging; Moderate patients have clinical symptoms (i.e. fever and respiratory tract symptoms) and pneumonia on chest imaging. Severe patients who meet any one of the following criteria: respiratory rate ≥30 breaths/minute; resting oxygen saturation ≤93%; arterial partial pressure of oxygen (PaO2)/oxygen concentration (FiO2) ≤300mmHg;

disease progression within 24 to 48 hours on chest image.

Supplementary Table S3. Details of studies on the COVID-19 vaccine coverage in the homeless population

Study	Country	Study period	Study design	Sample size	Category of Homeless*	Sex	Mean age (years)	Vaccine platforms	Vaccination coverage
**Balut et al, 2021 [26]**	USA	2020/12 ~2021/08	Cross-sectional	83528	Homeless veterans	Male: 90.4%<br>Female: 9.6%	NA	NA	45.8% received at least one vaccine dose
**Tucker et al, 2021 [37]**	USA	2021/03 ~2021/10	Cross-sectional	128	Sheltered homeless	Male: 68%<br>Female: 32%	18-25	NA	28.9% received at least one vaccine dose
**Montgomery et al, 2021 [30]**	USA	2020/12 ~2021/08	Cross-sectional	91906	Mixed population	NA	NA	NA	40.5% received at least one vaccine dose; 32.4% received two vaccine doses
**Sharif et al, 2022 [33]**	Canada	2020/12 ~2021/09	Cohort study	23247	Mixed population	Male: 63.5%<br>Female: 36.5%	43.2 (15.7)	mRNA or Viral vector	61.4% received one vaccine dose; 47.7% received two vaccine doses
**Bentivegna et al, 2022 [27]**	Italy	2021/06 ~2021/09	Cross-sectional	160	Mixed population	Male: 88.8%<br>Female: 11.2%	NA	NA	22.5% received one vaccine dose
**Berrou et al, 2022 [28]**	UK	2020/12 ~2021/05	Cross-sectional	199	Mixed population	NA	NA	NA	47.3% received one vaccine dose
**Rosen et al, 2022 [32]**	USA	2021/05 ~2021/11	Cross-sectional	4949	Mixed population	NA	NA	NA	40.6% received at least one vaccine dose
**Shearer et al, 2022 [34]**	USA	2021	Cross-sectional	13236	Mixed population	NA	NA	mRNA or Viral vector	33.7% received at least one vaccine dose
**Richard et al, 2022 [35]**	Canada	2021/06 ~2021/09	Cross-sectional	728	Mixed population	Male: 66.1%<br>Female: 33.9%	NA	mRNA or Viral vector	80.4% received at least one vaccine dose; 46.3% received at least

									two vaccine doses
**Nilsson et al, 2022 [31]**	Denmark	2020/12 ~2021/10	Cross-sectional	6689	Mixed population	NA	NA	mRNA or Viral vector	60.7% received one vaccine dose; 55.4% received two vaccine doses
**Meehan et al, 2022 [29]**	USA	2021.02	Cross-sectional	106	Sheltered homeless	Male: 58.5% Female: 41.5%	Median 44 (range: 18-70)	NA	43.4% received one vaccine dose; 17.0 % received two vaccine doses
**Rogers et al, 2022 [36]**	USA	2020-12~2021/02	Cross-sectional	672	Sheltered homeless	Male: 61.3% Female: 38.7%	Median 41	NA	0.6% received at least one vaccine dose

Abbreviation: NA: Not available.

*Mixed population means investigated subjects including both sheltered and unsheltered homeless.

Supplementary Figure S1

Study	Events	Total	Prevalence	[95%-CI]	Weight
**Sheltered homeless**					
Tobolowsky et al, 2020	35	195	0.18	[0.13; 0.24]	2.3%
Baggett et al, 2020	147	408	0.36	[0.32; 0.41]	2.4%
O'Shea et al, 2020	1	104	0.01	[0.00; 0.05]	2.2%
Mosites et al, 2020	293	1192	0.25	[0.22; 0.27]	2.5%
Karb et al, 2020	35	299	0.12	[0.09; 0.16]	2.4%
Imbert et al, 2020	101	150	0.67	[0.59; 0.74]	2.3%
Kelly et al, 2020	2	281	0.01	[0.00; 0.03]	2.4%
Gombita et al, 2020	0	331	0.00	[0.00; 0.01]	2.4%
Yoon et al, 2020	36	1690	0.02	[0.02; 0.03]	2.5%
Wang et al, 2020	372	10588	0.04	[0.03; 0.04]	2.5%
Ghinai et al, 2020	431	1435	0.30	[0.28; 0.32]	2.5%
Marquez et al, 2020	22	2456	0.01	[0.01; 0.01]	2.5%
Redditt et al, 2020	25	60	0.42	[0.30; 0.54]	2.0%
Jatt et al, 2020	0	121	0.00	[0.00; 0.03]	2.2%
Rogers et al, 2021	25	1275	0.02	[0.01; 0.03]	2.5%
Kiran et al, 2021	33	214	0.15	[0.11; 0.21]	2.3%
Husain et al, 2021	29	100	0.29	[0.21; 0.39]	2.2%
Ly et al, 2021	26	126	0.21	[0.14; 0.29]	2.3%
Ly et al, 2021	37	411	0.09	[0.07; 0.12]	2.4%
Kiran et al, 2021	80	1000	0.08	[0.06; 0.10]	2.5%
Roland et al, 2021	91	1985	0.05	[0.04; 0.06]	2.5%
Hsu et al, 2021	47	318	0.15	[0.11; 0.19]	2.4%
Lindner et al, 2021	0	51	0.00	[0.00; 0.07]	2.0%
Huggett et al, 2021	502	3657	0.14	[0.13; 0.15]	2.5%
Chang et al, 2022	193	322	0.60	[0.54; 0.65]	2.4%
Rowan et al, 2022	44	509	0.09	[0.07; 0.11]	2.4%
Bojorquez-Chapela et al, 2022	7	481	0.01	[0.01; 0.03]	2.4%
Berner et al, 2022	903	11563	0.08	[0.07; 0.08]	2.5%
**Random effects model**		**41322**	**0.11**	**[0.08; 0.15]**	**66.4%**
Heterogeneity: $I^2 = 99\%$, $p = 0$					
**Mixed population**					
Baggett et al, 2020	429	1297	0.33	[0.31; 0.36]	2.5%
Seballos et al, 2020	3	94	0.03	[0.01; 0.09]	2.2%
Baggio et al, 2021	69	215	0.32	[0.26; 0.39]	2.3%
Richard et al, 2021	274	8451	0.03	[0.03; 0.04]	2.5%
Oette et al, 2021	4	130	0.03	[0.01; 0.08]	2.3%
Ralli et al, 2021	82	960	0.09	[0.07; 0.10]	2.5%
Song et al, 2021	54	1717	0.03	[0.02; 0.04]	2.5%
Thomas et al, 2021	159	3153	0.05	[0.04; 0.06]	2.5%
Luong et al, 2022	394	4657	0.08	[0.08; 0.09]	2.5%
Morrone et al, 2022	242	6468	0.04	[0.03; 0.04]	2.5%
**Random effects model**		**27142**	**0.08**	**[0.05; 0.13]**	**24.2%**
Heterogeneity: $I^2 = 99\%$, $p < 0.01$					
**Unsheltered homeless**					
Yoon et al, 2020	3	636	0.00	[0.00; 0.01]	2.5%
Bihan et al, 2021	21	115	0.18	[0.12; 0.26]	2.2%
Fini et al, 2021	6	234	0.03	[0.01; 0.05]	2.4%
Rowan et al, 2022	6	239	0.03	[0.01; 0.05]	2.4%
**Random effects model**		**1224**	**0.04**	**[0.00; 0.11]**	**9.4%**
Heterogeneity: $I^2 = 95\%$, $p < 0.01$					
**Random effects model**		**69688**	**0.10**	**[0.08; 0.12]**	**100.0%**
Heterogeneity: $I^2 = 99\%$, $p = 0$					

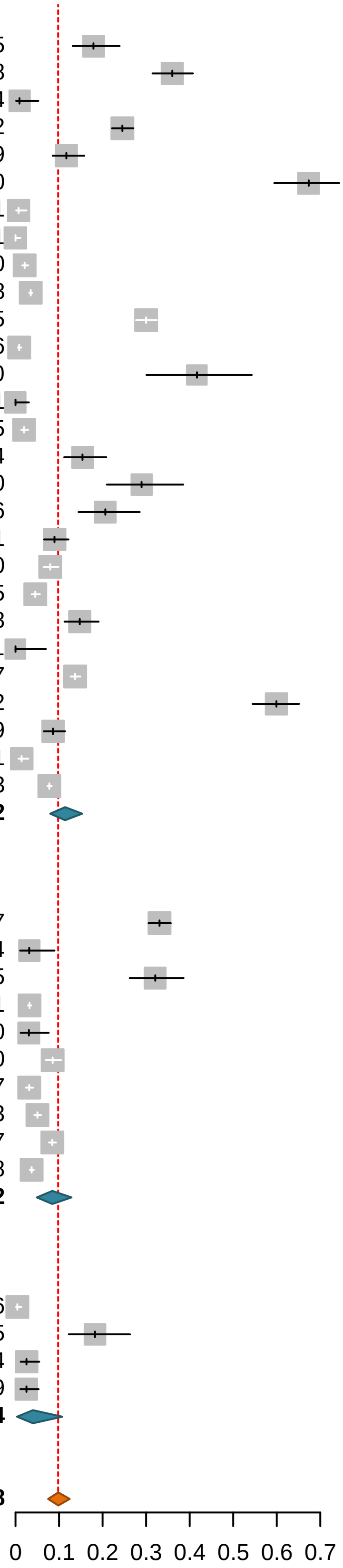

Checklist S1. Checklist of items to include when reporting a systematic review or meta-analysis

Section/topic	#	Checklist item	Reported paragraph # (within section)
**TITLE**			
Title	1	Identify the report as a systematic review, meta-analysis, or both.	Both
**ABSTRACT**			
Structured summary	2	Provide a structured summary including, as applicable: background; objectives; data sources; study eligibility criteria, participants, and interventions; study appraisal and synthesis methods; results; limitations; conclusions and implications of key findings; systematic review registration number.	Abstract was structured in format required by Journal
**INTRODUCTION**			
Rationale	3	Describe the rationale for the review in the context of what is already known.	1-3
Objectives	4	Provide an explicit statement of questions being addressed with reference to participants, interventions, comparisons, outcomes, and study design (PICOS).	2-3
**METHODS**			
Protocol and registration	5	Indicate if a review protocol exists, if and where it can be accessed (e.g., Web address), and, if available, provide registration information including registration number.	N/A
Eligibility criteria	6	Specify study characteristics (e.g., PICOS, length of follow-up) and report characteristics (e.g., years considered, language, publication status) used as criteria for eligibility, giving rationale.	1-2
Information sources	7	Describe all information sources (e.g., databases with dates of coverage, contact with study authors to identify additional studies) in the search and date last searched.	1-2

Section/topic	#	Checklist item	Reported paragraph # (within section)
Search	8	Present full electronic search strategy for at least one database, including any limits used, such that it could be repeated.	1
Study selection	9	State the process for selecting studies (i.e., screening, eligibility, included in systematic review, and, if applicable, included in the meta-analysis).	2
Data collection process	10	Describe method of data extraction from reports (e.g., piloted forms, independently, in duplicate) and any processes for obtaining and confirming data from investigators.	3
Data items	11	List and define all variables for which data were sought (e.g., PICOS, funding sources) and any assumptions and simplifications made.	3
Risk of bias in individual studies	12	Describe methods used for assessing risk of bias of individual studies (including specification of whether this was done at the study or outcome level), and how this information is to be used in any data synthesis.	4
Summary measures	13	State the principal summary measures (e.g., risk ratio, difference in means).	4
Synthesis of results	14	Describe the methods of handling data and combining results of studies, if done, including measures of consistency (e.g., I^2) for each meta-analysis.	4
Risk of bias across studies	15	Specify any assessment of risk of bias that may affect the cumulative evidence (e.g., publication bias, selective reporting within studies).	4
Additional analyses	16	Describe methods of additional analyses (e.g., sensitivity or subgroup analyses, meta-regression), if done, indicating which were pre-specified.	4
**RESULTS**			
Study selection	1	Give numbers of studies screened, assessed for eligibility, and included in the review, with reasons for	1, Fig. 1

Section/topic	#	Checklist item	Reported paragraph # (within section)
	7	exclusions at each stage, ideally with a flow diagram.	
Study characteristics	18	For each study, present characteristics for which data were extracted (e.g., study size, PICOS, follow-up period) and provide the citations.	2-3, Supplementary Table S1, S2 and S3
Risk of bias within studies	19	Present data on risk of bias of each study and, if available, any outcome-level assessment (see Item 12).	N/A
Results of individual studies	20	For all outcomes considered (benefits or harms), present, for each study: (a) simple summary data for each intervention group and (b) effect estimates and confidence intervals, ideally with a forest plot.	4-9, Fig. 2, Fig.3, Fig. 4, Fig. 5, Fig. 6, Fig. 7
Synthesis of results	21	Present results of each meta-analysis done, including confidence intervals and measures of consistency.	4-9, Fig. 2, Fig.3, Fig. 4, Fig. 5, Fig. 6, Fig. 7
Risk of bias across studies	22	Present results of any assessment of risk of bias across studies (see Item 15).	10
Additional analysis	23	Give results of additional analyses, if done (e.g., sensitivity or subgroup analyses, meta-regression [see Item 16]).	4
**DISCUSSION**			
Summary of evidence	24	Summarize the main findings including the strength of evidence for each main outcome; consider their relevance to key groups (e.g., health care providers, users, and policy makers).	1-4
Limitations	25	Discuss limitations at study and outcome level (e.g., risk of bias), and at review level (e.g., incomplete retrieval of identified research, reporting bias).	5
Conclusions	26	Provide a general interpretation of the results in the context of other evidence, and implications for future research.	6

Section/topic	#	Checklist item	Reported paragraph # (within section)
**FUNDING**			
Funding	27	Describe sources of funding for the systematic review and other support (e.g., supply of data); role of funders for the systematic review.	NA
